# 678. Changes in the *Clostridioides difficile* Infection (CDI) Treatment Pattern Pre-Post Clinical Guideline Update in 2021 and Variations across Hospitals

**DOI:** 10.1093/ofid/ofad500.740

**Published:** 2023-11-27

**Authors:** Erik R Dubberke, Qinghua Li, Engels N Obi, Vladimir Turzhitsky, Fakhar Siddiqui, Brian H Nathanson

**Affiliations:** Washington University, Saint Louis, Missouri; Merck & Co., Inc.,, Rahway, New Jersey; Merck & Co., Inc, BASKING RIDGE, New Jersey; Merck & Co., Inc., Boston, Massachusetts; Merck & Co., Inc., Boston, Massachusetts; OptiStatim LLC, Longmeadow, Massachusetts

## Abstract

**Background:**

In June 2021, IDSA updated its guideline to have fidaxomicin (FDX) as the preferred treatment over vancomycin (VAN) for patients with initial and recurrent CDI. The objective of this study was to examine changes in treatment pattern for CDI and variation in implementation of the guideline recommendation across hospitals.

**Methods:**

This was a retrospective, observational study using data from the PINC AI Healthcare Database, an electronic laboratory, pharmacy and billing data repository from about 25% of US hospitals. This study included patients aged 18 and older who received CDI treatment during the study period from 1/2020 to 6/2021 (pre-period) and from 10/2021 to 6/2022 (post-period). We first examined the treatment pattern in the pre- and post-periods by calculating the proportion of patients who received FDX on day one of treatment, only FDX, only VAN, only metronidazole (MTZ), switched from VAN or MTZ to FDX, and other treatment patterns. Then we examined proportion of patients receiving FDX on day one at the hospital level. We used a multilevel logistic regression model with hospital random effects to examine the association between hospital characteristics and FDX use. Patient and hospital characteristics were controlled.

**Results:**

67,311 CDI patients from 798 hospitals met the inclusion criteria. Figure 1 shows the analysis of CDI treatment pattern at the patient level. The proportion of patients treated with FDX on day one increased from 2.4% in the pre-period to 6.9% in the post-period. Patients who received only FDX increased from 1.3% to 4.1%, and those with a switch from VAN/MTZ to FDX from 3.2% to 6.4%. At the hospital level, the mean use of FDX on day one increased from 2.6% (SD = 4.8%) to 7.9% (SD = 10.1%), and the median increased from 1.1% (IQR= 0%, 3.4%) to 4.2% (IQR= 0%, 10.0%). We found that FDX use increased proportionately by all hospital characteristics except census region where greater increases were found in the South and Midwest. Hospitals had a statistically significant association with FDX use (p< 0.001) and 29.7% of the variance of FDX use on day one was explained by between-hospital variability.

**Figure:**
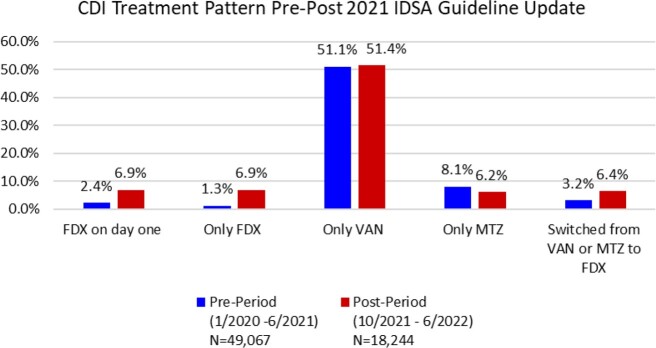

Adjusted Proportion of Treatment Starting with Fidaxomicin

**Figure:**
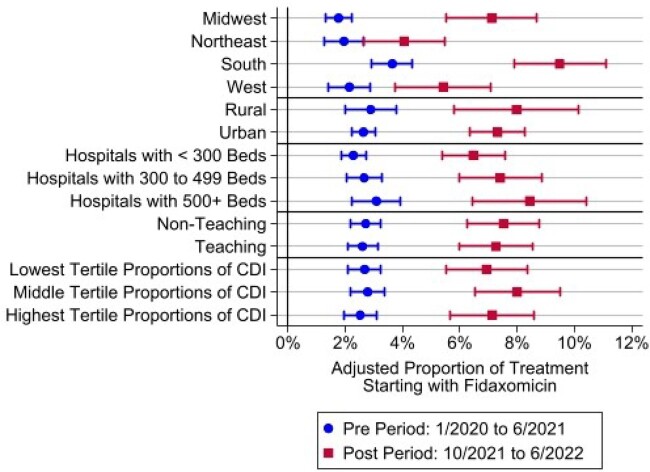

**Conclusion:**

Use of FDX for CDI treatment has increased since the publication of IDSA guideline in 2021, but it remains low with significant regional variation.

**Disclosures:**

**Erik R. Dubberke, MD, MSPH**, Abbott: Advisor/Consultant|AstraZeneca: Advisor/Consultant|Ferring Pharmaceuticals: Advisor/Consultant|Ferring Pharmaceuticals: Grant/Research Support|Merck and Co.: Advisor/Consultant|Pfizer: Advisor/Consultant|Pfizer: Grant/Research Support|Seres Therapeutics: Advisor/Consultant|Summit: Advisor/Consultant|Theriva Biologics: Grant/Research Support **Qinghua Li, PhD**, Merck & Co Inc: Employee **Engels N. Obi, PhD**, Merck & Co Inc: Employee **Vladimir Turzhitsky, PhD**, Merck & Co.: Full time employee of Merck & Co.|Merck & Co.: Stocks/Bonds **Fakhar Siddiqui, MD, MBA**, Merck & Co Inc.: Employee **Brian H. Nathanson, Ph.D.**, Merck & Co., Inc: Advisor/Consultant

